# Pattern of Prevalent Hepatitis B Virus Genotypes in Zaria, Nigeria

**DOI:** 10.4103/npmj.npmj_59_19

**Published:** 2019

**Authors:** Abdurrahman Elfulaty Ahmad, Adamu Girei Bakari, Bolanle Olufunke Priscilla Musa, Shettima Kagu Mustapha, Bello Yusuf Jamoh, Idris Nasir Abdullahi, Mohammed Ibrahim Tahir, Abdulqadri Olarenwaju Olatunji, Sumayya Hamza Maishanu, Ahmed Babangida Suleiman, Afolaranmi Tolulope, Claudia Hawkins, Atiene Solomon Sagay, Ayuba Zoakah, Adebola Tolulope Olayinka

**Affiliations:** 1 Department of Medicine, Ahmadu Bello University, Zaria, Nigeria.; 2 Department of Medical Laboratory Science, Ahmadu Bello University, Zaria, Nigeria.; 3 Research Unit, DNA LABS, Kaduna, Nigeria.; 4 Department of Microbiology, Ahmadu Bello University, Zaria, Nigeria.; 5 Department of Community Medicine, University of Jos, Jos, Nigeria.; 6 Department of Medicine, Division of Infectious Diseases, Feinberg School of Medicine, Northwestern University, Chicago, Illinois, USA.; 7 Department of Obstetrics and Gynaecology, University of Jos, Jos, Nigeria.; 8 Department of Medical Microbiology, Ahmadu Bello University, Zaria, Nigeria.

**Keywords:** Genotypes, hepatitis B Virus, mixed-infection, Nigeria, Zaria

## Abstract

**Background:**

Hepatitis B virus (HBV) is hyperendemic in Nigeria. Available literature reveal genotype E as being predominant in West Africa. This study aimed at identifying the current pattern and prevalent genotypes of HBV in Zaria, Nigeria.

**Materials and Methods:**

Four millilitre of blood was collected in ethylenediaminetetraacetic acid-container from each of 165 HBV surface antigen-positive participants recruited purposively from the gastroenterology clinic from May to August, 2017. Plasma was separated and frozen at −20°C till analysis. Multiplex-nested polymerase chain reaction using type-specific primers was used to identify the various HBV genotypes.

**Results:**

Median (and interquartile range ) age of the participants was 31.0 (25.5–39.0) years, with males constituting 107 (64.8%). Majority (83.6%) of the samples analysed were HBV-DNA-positive with 82.6% of the HBV-DNA-positive samples being mixed genotype infections. Irrespective of mode of occurrence, five HBV genotypes were identified with HBV/E (97.1%) being the most predominant, followed by HBV/B (82.6%), HBV/A (24.6%), then HBV/C (17.4%), while HBV/D (0.7%) was the least prevalent.

**Conclusion:**

In most (99.1%) of the mixed-infection were a combination of genotype E, the predominant genotype, with other genotypes predominantly genotype B. HBV genotypes E, B, A, C and D are the prevalent genotypes in Zaria, Nigeria, as they occur in single genotype and in mixed-genotypes pattern.

## Introduction

Hepatitis B virus (HBV) is one of the major causes of morbidity and mortality worldwide.^[1]^ About 2 billion people are thought to have evidence of the past or present infection with HBV, with about 240 million chronic carriers of HBV surface antigen (HBsAg).^[2]^ Worldwide, approximately 650,000 people die each year from complications of chronic hepatitis B.^[3]^ In Nigeria, HBV infection is hyperendemic with the seroprevalence of HBsAg ranging from 10% to 40%.^[4–8]^

Recently, ten genotypes of HBV (A through J) have been identified on the basis of 8% or less difference in genome sequences, each with a distinctive geographical distribution.^[9,10]^ In Sub-Saharan Africa, genotypes E is predominant in the region, followed by genotypes A and D.^[11,12]^ Different HBV genotypes present different clinical outcome and response to interferon-based therapy. Typically, infections with HBV genotypes A and D tend to progress to chronic phase than genotypes B and C, while genotypes A and B have higher rates of spontaneous HBeAg seroconversion compared to C and D.^[13]^ Genotype E, the most common HBV genotype in the West African region is associated with poor response to interferon-based therapy and is also moderately associated with pre-core and basal-core promoter mutations.^[14,15]^ Moreover, recent studies elsewhere show unusual HBV mixed genotype infections, suggesting overlapping clinical outcomes.^[16]^ In this study area, there is no prior study identifying the prevalent HBV genotypes and their pattern of infection. Therefore, this study aimed at identifying the current pattern and prevalent genotypes of HBV in Zaria, Nigeria.

## Materials and Methods

### Ethics statement

Ethical approval (dated: 25^th^ January, 2017; Reference Number: ABUTH/HREC/Y5/2016) was obtained from the Health Research Ethics Committee of Ahmadu Bello University Teaching Hospital, Zaria, before the commencement of sample collection. Written informed consent was sought and obtained from each participant prior to enrolment into the study as all the participants were adults. The participants were adequately informed of their right to choose to or not participate or withdraw at any point they so wished. All data were treated with the utmost confidentiality.

### Minimum sample size determination

The minimum sample size was calculated using single proportion formula:

$$N=Z^{2}Pq/d^{2}$$

where

*N* = Minimum sample size

*Z*^2^ = Standard Normal deviate set at 1.96.

*P* = Prevalence rate of 12.2% (0.122) was recorded according to Olayinka *et al*.^[8]^

*d* = acceptable error of 5% (0.05)

Therefore, the minimum sample size was calculated to be 165.

### Study area and subjects

This study was conducted in the gastroenterology clinic from May to August 2017. Zaria is a major city in Kaduna State of Northern Nigeria housing multiple Federal Government parastatals in addition to the study centre, such as the Ahmadu Bello University, the Nigerian College of Aviation Technology, the Nigerian Institute for Chemical and Leather Technology and Nigerian Institute for Transport Technology, among others. One hundred and sixty-five study participants with known chronic HBV infection were recruited purposively and had 4 mL of their venous blood samples collected. Chronic phase was defined by minimum of 6 months of infection obtained from their clinical records in the gastroenterology clinic.

### Sample collection and storage

For the analysis, the collected ethylenediaminetetraacetic acid-anticoagulated blood was centrifuged at 2800 × xg for 5 min to separate the plasma. All samples collected were first tested immunochromatographically for HCV and HIV for exclusion, then reconfirmed for HBsAg status using FaStep rapid immunochromatographic test strip (Polymed Therapeutics, Inc. Houston, USA). The separated plasma was then transferred into cryovials and stored at −20°C till analysis. All the plasma were separated in Immunology Laboratory of the Teaching Hospital and transported in cold chain to DNA LABS Kaduna, Nigeria, where Hepatitis B viral genotyping was conducted.

### Hepatitis B virus DNA detection and genotyping by nested polymerase chain reaction

Multiplex-nested polymerase chain reaction (PCR) using type-specific primers [Table 1] was used to assign genotypes A through F based on pre-S1 through S genes of the HBV genome. The design of the HBV genotype-specific primers is based on the conserved regions of the sequences in a particular genotype and discordance in homology with the sequences of other HBV genotypes.^[17]^ Five such different sets of primers were used: P_1_ and S_1–2_ being the universal outer primers and B2 was used as the inner sense (forward) primer with a combination of BA1R, BB1R and BC1R as anti-sense (reverse) inner primers for genotypes A, B and C, respectively, in a multiplex system tagged ‘Mixture A’. For genotypes D, E and F, an anti-sense primer B2R was used in combination with BD1, BE1and BF1 as sense (forward) primers, also in a multiplex system tagged ‘Mixture B’. List and classification of primers used are shown in Table 1.

### Hepatitis B virus DNA extraction

HBV DNA was extracted from plasma of HBsAg-positive participants using the Bioneer *AccuPrep* Genomic DNA extraction kit according to the manufacturer’s instructions.

### First-round polymerase chain reaction: Hepatitis B virus DNA detection

The final reaction volume for the first round of the nested PCR was 20 μL. The premix tubes were labelled with the sample ID. Two microlitres of extracted DNA was added to a Master Mix (cocktail of 16 μL of deionised water [D.H_2_O] and premix of 250 μM of each dNTP, 1X PCR buffer, 15 mM of MgCl_2_ and 1U of thermostable Taq polymerase) and 1 μL each of P_1_ (forward) and S_1–2_ (reverse) outer primers. The PCR was performed using thermal cycler (PTC-100™ Programmable thermal controller, MJ Research, Inc.) and reaction conditions were set as: initial activation at 95°C for 5 min; denaturation at 94°C for 20 s; annealing at 55°C for 20 s and extension at 72°C for 1 min. Complete sets of 30 cycles from denaturation to extension were observed. Then, the final extension was set at 72°C for 5 min.

### Second-round polymerase chain reaction: Hepatitis B virus genotyping

Second-round PCR was performed in two different tubes for each sample, one with the common universal sense primer (B2) and type-specific primers for genotypes A, B, C in ‘Mixture A’ and the other with the common universal anti-sense primer B2R and type-specific primers for genotypes D, E, F in ‘Mix-B’. Seventeen microliters of D.H_2_O was added into each tube of premix ‘A’ and ‘B’. Two microlitres of the cocktail of primers (containing 0.5 μL each of the four primers) were added into the mixtures. One microliter of the first-round PCR product into each tube of the premix. The mixture was mixed gently and centrifuged. The PCR condition was set as: initial activation at 94°C for 3 min, followed by 30 cycles of denaturation at 94°C for 1 min, annealing at 50°C for 1 min and extension at 72°C for 1 min for both ‘Mix A’ and ‘Mix B’, with the final extension at 72°C for 5 min. Primers used were adopted from previous studies.^[18, 19]^ Twenty microliters of each of negative control, samples and the ladder were run on 2% agarose gel (2% w/v in 1 × TAE buffer) and electrophoresed in 1 × TAE buffer for 45 min at 100V. The bands were visualised under gel documentation system (BioRad Gel Doc-XR, USA) and screenshots captured. The size of the separated bands (DNA fragments) was compared with the GeneRuler™ 100 bp + DNA ladder (MBI Fermentas, Life Sciences, Canada). We deposited the detailed procedure we used for the HBV genotyping on protocols.io.^[20]^

### Statistical analysis

The data were collated and validated using Epi Info ® questionnaire database (CDC, Atlanta, Georgia, USA). It was then analysed using GraphPad Prism 6 statistical software package (GraphPad Software, Inc. San Diego, California, USA). Frequencies and percentages of the identified genotypes were presented in tables and charts, while age was presented as median (and interquartile range  [IQR]). Chi-squared test with Yates correction was used to determine the relationship between identified HBV genotypes and sex and age groups.

## Results

### Sociodemographic characteristics

The median (and IQR) age of the 165 HBsAg-positive participants was 31.0 (25.5–39.0) years. Male participants constituted 64.8% (107/165) of the total number recruited. Age group of 18–27 years constituted the highest number of participants with 62 (37.6%), which was followed by the 28–37 year group with 55 (33.3%), then the 38–47 and 48–57-year groups with 33 (20.0%) and 15 (9.1%), respectively. Those with tertiary education constituted the majority of the participants with 80 (48.5%) and were followed by those in the secondary level with 46 (27.6%), those with only Qur’anic/Islamic education with 17 (10.3%), those in post-graduate level with 12 (7.3%), then those with only primary education with 9 (5.5%) and only 1 (0.6%) individual without any form of education. Civil servants were the highest number of participants with 49 (29.7%), followed by the self-employed, students, homemakers, others, non-governmental employees and retirees with 40 (24.2%), 38 (23.0%), 30 (18.2%), 4 (2.4%), 3 (1.8%) and 1 (0.6%), respectively. Ninety-nine (60%) of the study participants were married men and women, and this was followed by singles with a frequency of 59 (35.8%). Widowed women and divorced participants had a frequency of 4 (2.4%) and 3 (1.8%), respectively. All these characteristics and distributions are as shown in Table 2.

**Figure 1:**
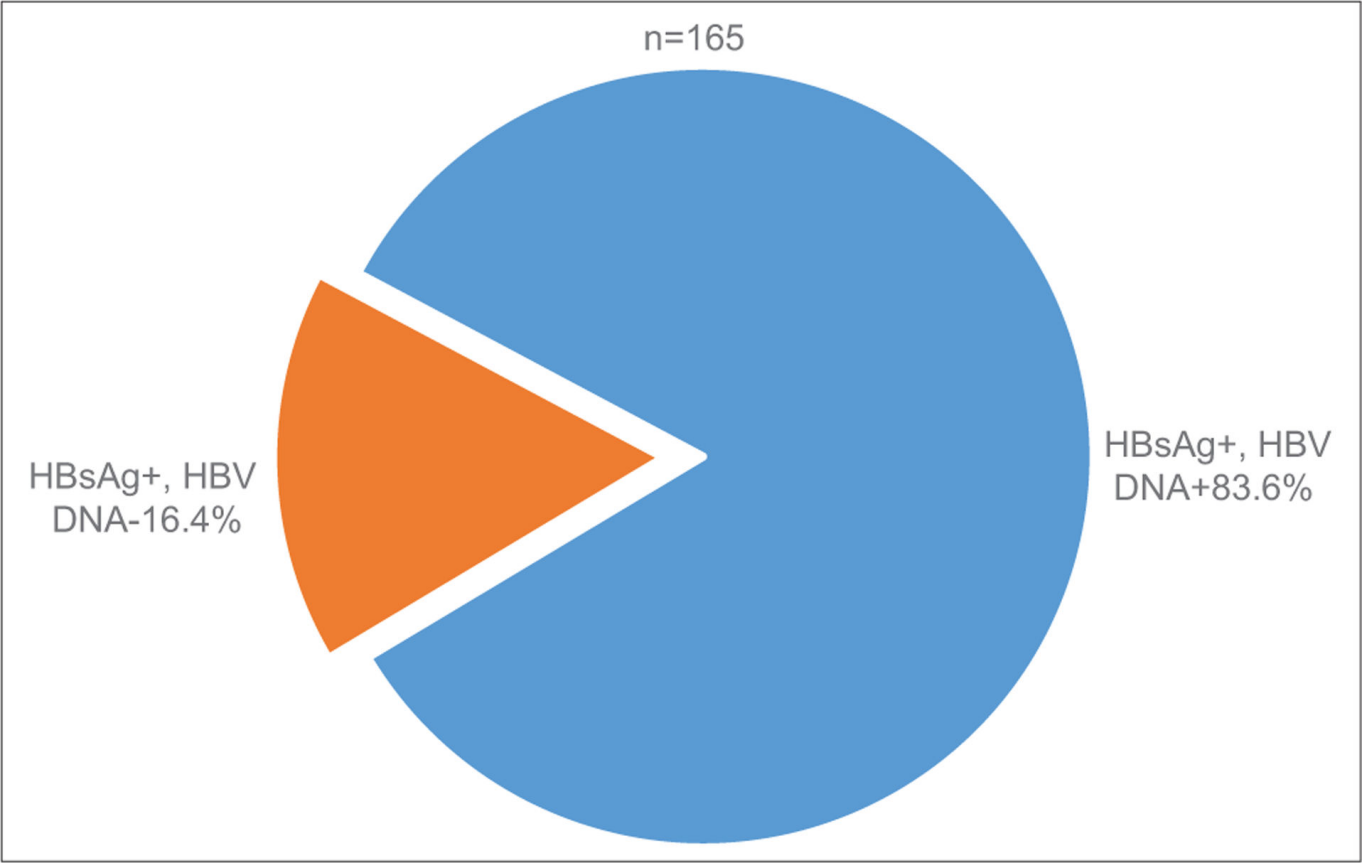
Proportion of HBV surface antigen-positive participants with HBV-DNA positivity and negativity by primer-specific nested polymerase chain reaction. A pie chart showing a greater proportion of the HBV-DNA-positive samples (138/165). HBV: Hepatitis B virus

**Figure 2:**
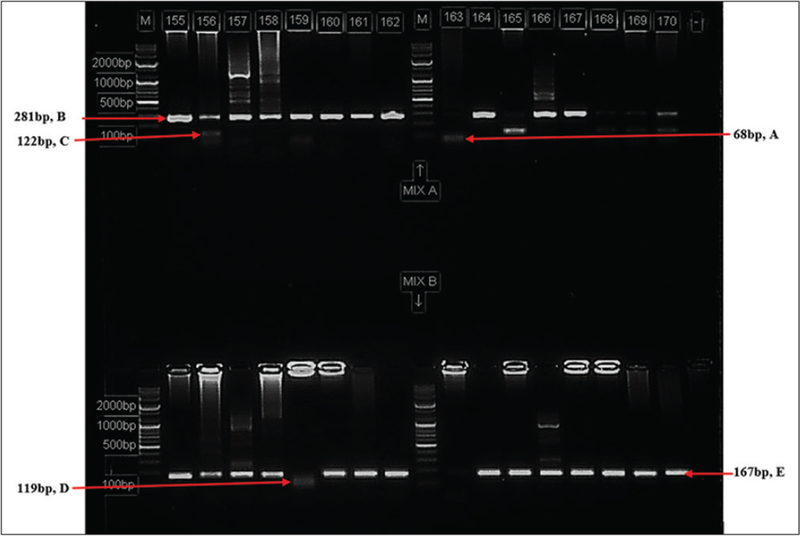
Agarose gel electrophoretogram of HBV surface antigen-positive HBV DNA+ samples. Mix A and B identifying HBV genotypes A (68bp), B (281bp), C (122bp), D (119bp) and E (167bp). M: 100bp+ molecular marker; (−): Negative control; Representative samples 155–170 samples. HBV: Hepatitis B virus

**Figure 3:**
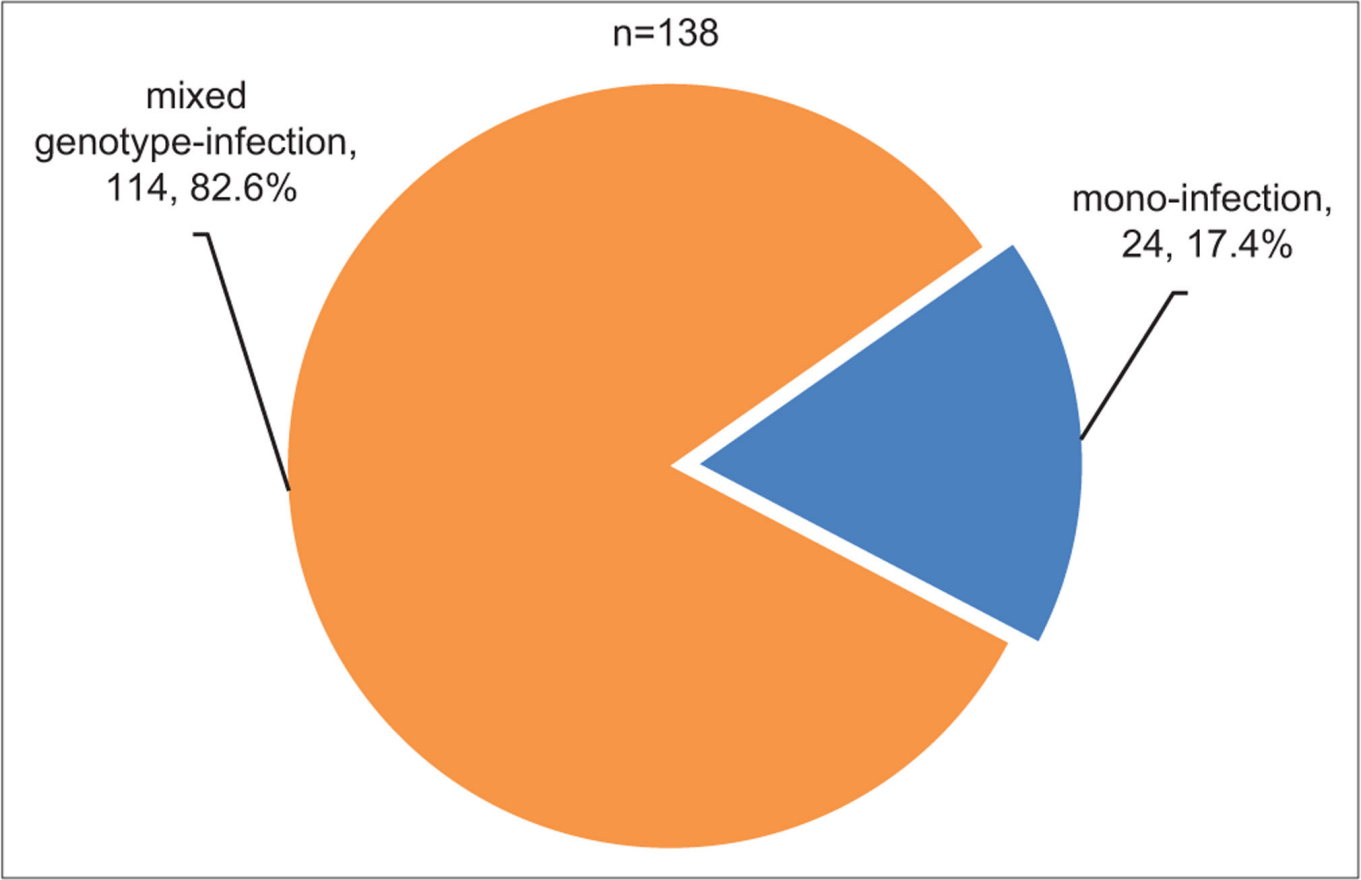
Pattern of HBV infection in Zaria. Pie chart showing proportions of mono- and mixed-infections with HBV in HBV-DNA-positive patients in Zaria, Nigeria. HBV: Hepatitis B virus

**Figure 4:**
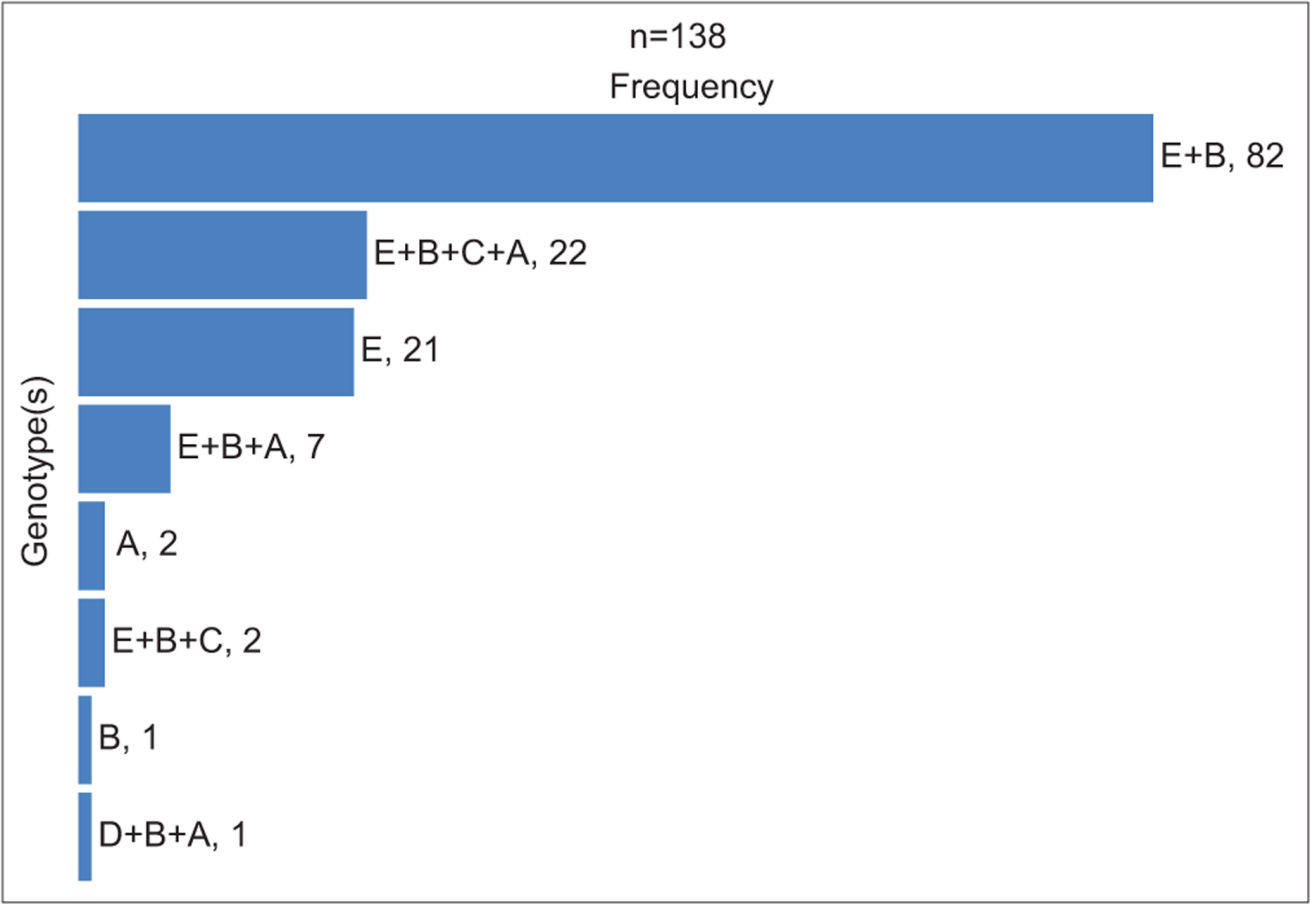
Hepatitis B virus genotypes distribution in HBV-DNA-positive samples in Zaria. A bar chart showing HBV genotypes in mono- and mixed-infection patterns prevalent in Zaria, Nigeria. HBV: Hepatitis B virus

**Figure 5:**
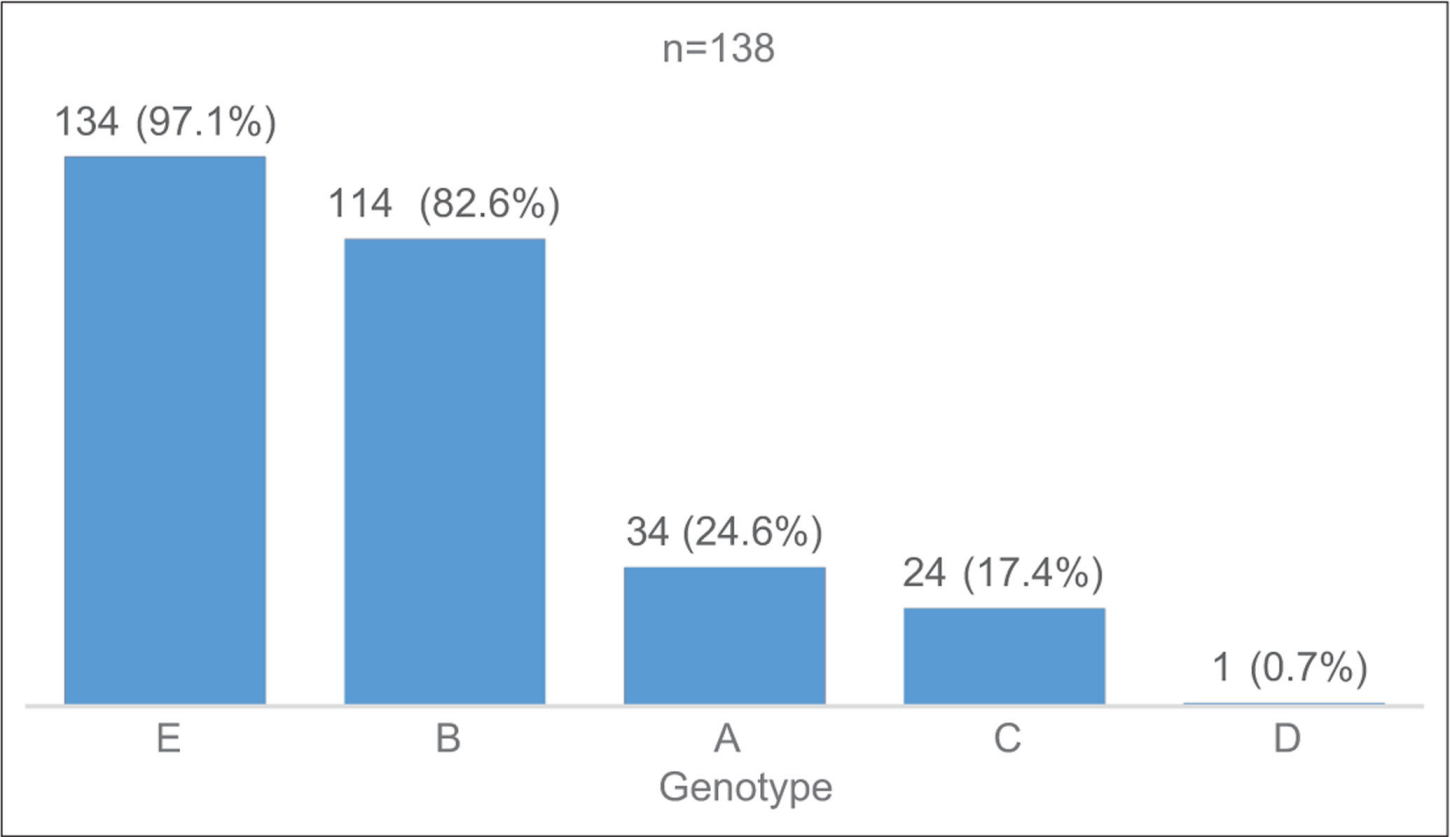
HBV single genotypes distribution in HBV-DNA-positive samples in Zaria. A column chart showing the prevalence of HBV genotypes in Zaria, Nigeria. HBV: Hepatitis B virus

Out of the 165 HBsAg-positive samples collected, 138 (83.6%) were HBV-DNA positive [Figure 1]. Electrophoretogram of samples from HBsAg-positive HBV-DNA-positive study participants show bands representing identified genotypes. Bands from Mix ‘A’ of the primers set-up were shown at the upper part of the gel, while bands from Mix ‘B’ were shown at the lower part of the gel. Genotypes A, B and C with band sizes of 68 bp, 281 bp and 122 bp, respectively, were identified in Mix ‘A’, while genotypes D and E with band sizes of 119 bp and 167 bp, respectively, were identified in Mix ‘B’. Molecular marker (M) of 100 base pairs plus (100 bp+) served as the marker for identifying the genotypes of the HBV. Sample #155 showed mixed genotype infection with genotypes B and E; sample #156 had mixed genotypes B, C and E; sample #159 showed mixed genotypes of B and D; while sample #163 showed mixed genotypes of A and E [Figure 2]. Out of the 138 HBsAg-positive HBV-DNA-positive samples, 114 (82.6%) had mixed HBV genotypes infection, while the remaining 24 (17.4%) had single-genotype infection [Figure 3]. With respect to the pattern of infections, genotypes E + B mixed-infection had the highest frequency of 82 (59.5%), while the least frequent were genotype B mono-infection and genotypes D+B+A mixed-infection each with 1 (0.7%) [Figure 4]. Stratifying the HBV genotypes by age group and sex, there was no significant association between either the age group and HBV genotypes (*P* = 0.8024) or sex and the HBV genotypes (*P* = 0.7501) [Table 3]. For the HBV-DNA-positive samples analysed for genotyping, genotype E was most prevalent with 134 (97.1%), while genotype D had 1 (0.7%) [Figure 5].

## Discussion

In Nigeria, most of the studies conducted on HBV were restricted to the prevalence of the virus in different population subgroups such as blood donors,^[21]^ pregnant women and children,^[22,23]^ and some focused on a meta-analysis which pooled the different subgroups to arrive at some figures.^[24]^ A recent national survey focusing on the prevalence and risk factors associated with HBV infection^[8]^ is also available, the final findings of which informed the sample size used for this study. Hepatitis B infection is a vaccine-preventable disease, the vaccine which was included in the Nigerian national immunisation schedule in 1995 only to be available in 2004.^[22,25,26]^ The participants in this studyhad a median age of 31.0 (25.5–39.0) years and a minimum age of 18 years did not have the privilege of getting the shots of the hepatitis B vaccines at birth, as they were all born before the inclusion of the vaccine in the national immunisation schedule. The study showed there were more male participants with HBV as compared to the female participants. This agreed with other previous studies.^[16,17]^ The underlying higher prevalence of HBV among men in Africa could explain the male predominant cohort.

Our study was able to detect and genotype the infecting HBV in 83.6% (138/165) of the collected samples. This was consistent with the findings in Egypt^[27]^ using innogenetics line probe assay (INNO-LiPA) which is based on the reversed hybridisation principle; in the United Arab Emirates using the nucleic acid testing (NAT);^[28]^ in Cote d’Ivoire using nested PCR;^[17]^ in India,^[29]^ using the NAT and in Wasit province of Iraq^[16]^ using nested PCR where they were able detect and genotype 71.4%, 95%, 68.7%, 69.7% and 60.5%, respectively. Similarly, much earlier, using mini-pool and single-sample PCR assays, another study^[30]^ detected HBV-DNA in 69% of HBsAg-positive samples. The different methodologies applied maintained the variability in HBV-DNA isolation in relation to HBsAg positivity, in that not all HBsAg-positive samples yielded positive results for HBV-DNA detection, irrespective of the method of detection. Being a non-encapsulated virus, HBV-DNA tends to rapidly degrade, whereas in the absence of or with inadequate anti-HBs, viral surface antigen may remain in circulation for a prolonged periods of time.^[31]^ This could depend on the stage of infection such as in patients who are chronic carriers with inactive infection. It could also be explained by intermittent viraemia or an extremely low and undetectable levels of HBV-DNA either due to prior treatment or natural clearance.^[32]^

Mixed genotype infection was shown to dominate over single genotype infection with 82.6% (114/165) as against the latter with 17.4% (24/165). This agrees with the findings in Iraq,^[33]^ where they documented all 4 HBV-DNA-positive samples to have mixed genotype infections with 75% (3/4) harbouring 4 different genotypes and 25% (1/4) harbouring 3. It is also in agreement with the findings in Iraq,^[16]^ where they identified mixed genotype infection in all the samples, recording not a single sample with single genotype infection. Our findings of mixed genotype infection constituting the better portion, however, disagrees with that of a finding in Taiwan,^[34]^ where mono-infection with genotype B was identified to predominate. It also disagrees with a study in Paris^[35]^ where in comparing direct sequencing with INNO-LiPA HBV genotyping assay, 85.3% was found to harbour a single genotype. The mixed-infection recorded in our study could be as a result of possible recombination between genotypes as noted in earlier findings.^[36–38]^ It could also be as a result of migrations and long-term travels for overseas studies and peacekeeping missions by military personnel.

In all, the identification of the five HBV genotypes in this study, namely, A, B, C, D and E corroborates the assertions^[39,40]^ that all of them are more frequently detected in Africa. Regardless of occurrence in the form of mixed or mono-infection, this study identified genotype E to be with the highest prevalence. This was followed by genotype B, then genotypes A, then C and genotype D with the least prevalence. However, where occurring as mono-infection, HBV/E still predominates with HBV/A being the second most prevalent with HBV/B being the least. Mixed-infection of HBV/E+B has the highest occurrence in all, which was followed by HBV/E+B+C+A, HBV/E+ B + A and HBV/E+B+C with the least mixed-infection being HBV/D+B+A. Available literature have proclaimed genotype E to be the predominant genotype in Nigeria^[11,41,42]^ and the Sub-Saharan Africa in general.^[43–45]^ This has confirmed the inclusion of Nigeria in the crescent of HBV genotype E in Sub-Saharan Africa from Senegal in the West to the Central African Republic in the East and Namibia in the South.^[39,46,47]^ It is a common belief that genotype E is almost exclusively found only in Africa, with few exceptions on other continents with links to Africans.^[48]^ This study is in agreement with the previous studies that showed E to be the most prevalent genotype in West Africa with genotype A being the second most prevalent, both having originated from the region.^[48]^ Although there was no specific mention of the prevalence rate of HBV/B in Nigeria or any West African country, it is believed that it is detected in Africa.^[40]^ Genotype B identified in this study appears to be favoured by prior infection of another genotype where it counts as a super-infection or has high recombination tendencies, considering that it is the second most prevalent genotype. Genotype D that was the least identified genotype is not unexpected as the previous studies have identified genotype D6 subtype *ayw2* in Nigeria.^[49]^

## Conclusion

HBV infection in Zaria is mostly characterised by mixed genotype infections. Irrespective of occurrence as mixed- or mono-infection, five genotypes of HBV are prevalent in Zaria, Nigeria, with HBV/E (97.1%) predominating, followed by HBV/B (82.6%), HBV/A (24.6%), HBV/C (17.4%) and HBV/D (0.7%) having the least occurrence. Most of the mixed-infection are a combination of genotype E, the predominant genotype, then other genotypes predominantly genotype B that appear to co- or super-infect.

### Limitation

The multiplex nested PCR method applied for genotyping HBV is capable of identifying only six genotypes; A-F, limiting this study to identifying only those. Genotypes G-J might have been missed by this method.

## Figures and Tables

**Table 1:** Sequences of primers for hepatitis B virus amplification and genotyping used by multiplex-nested polymerase chain reaction

Primer	Sequence (5’–3’)	Specificity	Position	Polarity
1^st^ round PCR				
PI	TCACCATATTCTTGGGAACAAGA	Universal	2823–2845	Sense
Sl-2	CGAACCACTGAACAAATGGC	Universal	685–704	Antisense
2^nd^ round PCR: Mix A				
B2	GGCTCCAGTTCCGGAACAGT	Type A-E	67–86	Sense
BA1R	CTCGCGGAGATTGACGAGATGT	Type A	113–134	Antisense
BB1R	GGTCCTAGGAATCCTGATGTTG	TypeB	165–186	Antisense
BC1R	CAGGTTGGTGAGCTGGAGA	TypeC	2979–2996	Antisense
2^nd^ round PCR: Mix B				
B2R	GGAGGCGGATTTGCTGGCAA	Type D-F	3078–3097	Antisense
BD1	GCCAACAAGGTAGGAGCT	Type D	2979–2996	Sense
BE1	CACCAGAAATCCAGATTGGGACCA	Type E	2955–2978	Sense
BF1	GTTACGGTCCAGGGTTACCA	Type F	3032–3051	Sense

**Table 2:** Sociodemographic characteristics of hepatitis B virus surface antigen-positive study participants in Zaria, Nigeria (*n*=165)

Variable	Frequency	(%) 95% Cl
Median age (IQR), years	31.0 (25.5–39.0)	
Gender		
Male	107	64.8 (57.0–72.1)
Female	58	35.2 (27.9–43.0)
Age group (years)		
18–27	62	37.6 (30.2–45.4)
28–37	55	33.3 (26.2–41.1)
38–47	33	20.0 (14.2–26.9)
48–57	15	9.1 (5.2–14.6)
Level of education		
Primary	9	5.5 (2.5–10.1)
Secondary	46	27.9 (21.2–35.4)
Tertiary	80	48.5 (40.6–56.4)
Post-graduate	12	7.3 (3.8–12.4)
Qur’anic/Islamiyya only	17	10.3 (6.1–16.0)
None	1	0.6 (0.01–3.3)
Occupation		
Civil servant	49	29.7 (22.8–37.3)
Self-employed	40	24.2 (17.9–31.5)
Non-governmental employee	3	1.8 (0.4–5.2)
Retired	1	0.6 (0.02–3.3)
Student	38	23.0 (16.8–30.2)
Homemaker	30	18.2 (12.6–24.9)
Others	4	2.4 (0.7–6.1)
Marital status		
Single	59	35.8 (28.5–43.6)
Married	99	60.0 (52.1–67.5)
Divorced	3	1.8 (0.4–5.2)
Widowed	4	2.4 (0.7–6.1)

Univariate analysis showing the frequency and percentages with 95% CI.

CI: Confidence interval, IQR: Interquartile range

**Table 3:** Sex and age stratification for the distribution of hepatitis B virus genotypes in Zaria, Nigeria

Variable	HBV/A (%)	HBV/B (%)	HBV/C (%)	HBV/D (%)	HBV/E (%)	Statistics (Yates*χ*^2^, df, *P*)
Age range						
18–27	17 (12.3)	42 (30.4)	11 (8.0)	0 (0.0)	50 (36.2)	7.775, 12, 0.8024
28–37	9 (6.5)	39 (28.3)	8 (5.8)	0 (0.0)	45 (32.6)	
38–47	7 (5.1)	23 (16.7)	4 (2.9)	1 (0.7)	28 (20.3)	
48–57	1 (0.7)	10 (7.2)	1 (0.7)	0 (0.0)	11 (8.0)	
Total	34 (24.6)	114 (82.6)	24 (17.4)	1 (0.7)	134 (97.1)	
Sex						
Male	19 (13.8)	76 (55.1)	15 (10.9)	1 (0.7)	85 (61.6)	1.922, 4, 0.7501
Female	15 (10.9)	38 (27.5)	9 (6.5)	0 (0.0)	49 (35.5)	
Total	34 (24.6)	114 (82.6)	24 (17.4)	1 (0.7)	134 (97.1)	

HBV: Hepatitis B virus
